# Rapid On-Site Microscopy and Mapping of Diagnostic Biopsies for See-And-Treat Guidance of Localized Prostate Cancer Therapy

**DOI:** 10.3390/cancers15030792

**Published:** 2023-01-27

**Authors:** Madeline R. Behr, Shams K. Halat, Andrew B. Sholl, Louis Spencer Krane, Jonathan Quincy Brown

**Affiliations:** 1 Department of Biomedical Engineering, Tulane University, New Orleans, LA 70118, USA; 2 Department of Pathology and Laboratory Medicine, Tulane University School of Medicine, New Orleans, LA 70112, USA; 3 Department of Pathology, Touro Infirmary, New Orleans, LA 70115, USA; 4 Department of Urology, Tulane University School of Medicine, New Orleans, LA 70112, USA; 5 Department of Urology, Southeast Louisiana Veterans Health Care System, New Orleans, LA 70112, USA

**Keywords:** microscopy, prostate cancer, localized therapy, fluorescence, biopsy, diagnosis

## Abstract

**Simple Summary:**

Localized ablation is an emerging treatment for prostate cancer that minimizes recovery time and improves functional retention. However, standard histological processing of diagnostic prostate biopsies is currently performed too late to inform the treatment of localized lesions at the time of the diagnostic biopsy procedure. We investigated the feasibility of structured illumination microscopy (SIM) as an adjunct tool to confirm malignancy in fresh prostate biopsies for a future “see-and-treat” paradigm of localized prostate cancer. Forty-six biopsies were assessed by two pathologists, resulting in 92% and 87% overall diagnostic accuracies, respectively. In a proposed “clinical decision tree”, urologist predictions from MRI fusion images were compared to diagnoses from SIM images, which demonstrated that SIM feedback would constructively inform on-site intervention of localized prostate cancer and confirm margins. We demonstrated that the process necessary to generate and diagnose pseudo-hematoxylin and eosin biopsy images via SIM can be achieved in well under 15 minutes.

**Abstract:**

Prostate cancer continues to be the most diagnosed non-skin malignancy in men. While up to one in eight men will be diagnosed in their lifetimes, most diagnoses are not fatal. Better lesion location accuracy combined with emerging localized treatment methods are increasingly being utilized as a treatment option to preserve healthy function in eligible patients. In locating lesions which are generally <2cc within a prostate (average size 45cc), small variance in MRI-determined boundaries, tumoral heterogeneity, patient characteristics including location of lesion and prostatic calcifications, and patient motion during the procedure can inhibit accurate sampling for diagnosis. The locations of biopsies are recorded and are then fully processed by histology and diagnosed via pathology, often days to weeks later. Utilization of real-time feedback could improve accuracy, potentially prevent repeat procedures, and allow patients to undergo treatment of clinically localized disease at earlier stages. Unfortunately, there is currently no reliable real-time feedback process for confirming diagnosis of biopsy samples. We examined the feasibility of implementing structured illumination microscopy (SIM) as a method for on-site diagnostic biopsy imaging to potentially combine the diagnostic and treatment appointments for prostate cancer patients, or to confirm tumoral margins for localized ablation procedures. We imaged biopsies from 39 patients undergoing image-guided diagnostic biopsy using a customized SIM system and a dual-color fluorescent hematoxylin & eosin (H&E) analog. The biopsy images had an average size of 342 megapixels (minimum 78.1, maximum 842) and an average imaging duration of 145 s (minimum 56, maximum 322). Comparison of urologist’s suspicion of malignancy based on MRI, to pathologist diagnosis of biopsy images obtained in real time, reveals that real-time biopsy imaging could significantly improve confirmation of malignancy or tumoral margins over medical imaging alone.

## 1. Introduction

Prostate cancer is the second most diagnosed malignancy in men with almost 270,000 new cases expected in America in 2022 [1]. Suspicion of prostate cancer is typically triggered by an abnormal prostate-specific antigen (PSA) test or digital rectal exam (DRE). After an abnormal result, magnetic resonance imaging (MRI) is often scheduled to determine whether clinically significant cancer is developing within the prostate. However, up to 20% of clinically significant lesions are not visible on MRI [2]. Based on the probability of cancer detection through the prostate imaging reporting and data system (PIRADS) scoring system, many patients will proceed with invasive testing via biopsy. Diagnostic prostate biopsies are performed either transrectally or transperineally, and increasingly with MRI/ultrasound (US) fusion guidance. During the procedure, two types of biopsies are taken: lesion biopsies which are collected from the region of interest (ROI) and systematic biopsies which are spaced throughout the prostate. Currently, no therapeutic procedures are performed until the final pathologic diagnosis is completed after formalin fixation and standard histological workup. 

Post-diagnosis, the patient’s treatment is based on several key factors, most importantly the Gleason score [3]. Most patients who undergo the biopsy procedure will have clinically significant cancer detected and are recommended for a curative local therapy. These range from surgical (radical prostatectomy) to radiotherapy (external beam radiotherapy, brachytherapy) to ablative (high-intensity focused ultrasound, cryoablation). The selected treatment method is based on a number of considerations: tumor aggression (via Gleason grade), patient functional status, lesion location, prostate dimensions, and patient life expectancy. For localized ablative therapies, the management of prostate cancer can be improved by minimizing the delay between the biopsy diagnosis and treatment of the lesion, potentially enabling a “see-and-treat” paradigm or real-time feedback of tumoral margin, which minimizes the risk of cancer spread, improves efficacy of tumor destruction, and limits overall patient discomfort. The intraoperative image guidance typically gives the clinician access to a 3D image representation of the needle tracks as well as MRI fusion images along the coronal, sagittal, and transverse planes during the biopsy procedure. However, the resolution for these images is limited since they are taken at a whole-organ level. This resolution is sufficient to guide biopsy locations, but not high enough to confirm malignancy or lesion margin information. Currently, it is not possible to treat localized lesions during the initial diagnostic biopsy procedure because of the lack of available histopathology information to confirm malignant prostate tissue on-site. Existing on-site histopathology methods including frozen section analysis (FSA) and touch-prep cytology (TPC) are not accurate enough to inform treatment decisions in prostate cancer [4,5].

Ex vivo microscopy is a set of emerging advanced optical sectioning microscopy technologies that are suitable for enabling histological imaging of non- or minimally processed tissues at the point of care [6]. These technologies include confocal microscopy [7,8,9,10,11,12,13,14,15], multiphoton microscopy [16], light sheet microscopy [17], microscopy with UV surface excitation (MUSE) [18,19,20], optical coherence imaging [21], non-linear optical imaging (NLOI) [22], and structured illumination microscopy [23,24,25,26,27,28]. Optical clearing methods have proven to be a useful tool for histological imaging with optical sectioning microscopies. Optical clearing and confocal microscopy have been combined to create 3D virtual H&E stacks from fresh samples from radical prostatectomies in about 30 h [29]. Optical clearing has also been harnessed in conjunction with open-top light-sheet microscopy to generate a grayscale histology-analogous image of prostate biopsies within one hour of biopsy acquisition [30]. Ex vivo microscopy has been particularly relevant in the advancement of prostate cancer research in improving both the prostate biopsy procedure and to guide radical prostatectomies. Confocal microscopy combined with fluorescence microscopy to image prostate biopsies for intraoperative analysis has been previously demonstrated in the literature [31,32,33,34]. Additionally, microscopy techniques have been investigated to overcome the delays incurred by standard histological workup or frozen sectioning in the assessment of surgical margins for radical prostatectomies [35,36,37].

Our group has advanced structured illumination microscopy (SIM) as a rapid and non-destructive option for ex vivo imaging. SIM is a wide-field optical sectioning method utilizing patterned illumination to reject out-of-focus light. SIM achieves comparable optical sectioning and imaging depth to confocal microscopy, but offers a speed advantage due to the utilization of wide-field imaging and its corresponding larger field of view [23]. SIM has been successfully utilized to image multiple tissue types including prostate, breast, kidney, and liver [24,25,26,27], including work to image and resolve clinically relevant information from previously frozen large-core prostate biopsies [25] and whole prostatectomy resection surfaces [38,39].

**Figure 1:**
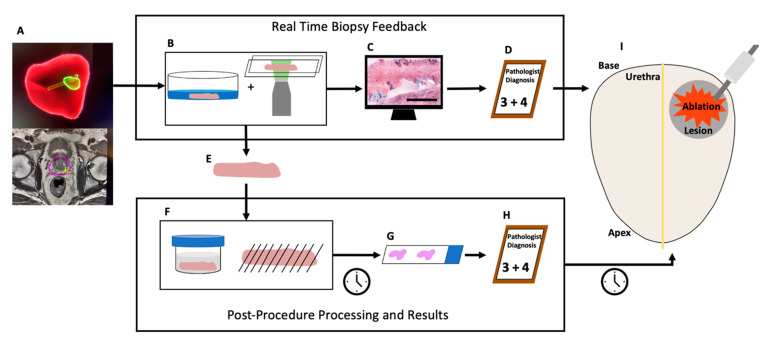
Potential “see-and-treat” localized prostate ablation informed by real-time SIM imaging compared to standard workflow. In this work, we assessed, retrospectively, the potential for SIM to provide real-time confirmation of lesion status during MRI-US-guided biopsy. A patient with a potentially malignant lesion with undefined boundaries enters the clinic and undergoes an MRI fusion transrectal diagnostic biopsy (**A**). SIM staining and imaging are performed immediately after excision (**B**). A digital H&E analog is generated (**C**, scale bar represents 1 mm). This image is presented to a pathologist for diagnosis (**D**). The same biopsy is transported to histology (**E**) where it undergoes traditional processing (**F**) and is mounted on a slide (**G**). The histology slide is used by a pathologist to make a diagnosis (**H**). A malignant diagnosis with a Gleason grade of 3 + 4 would result in localized ablation therapy (**I**). This therapy is currently delayed by histological processing and follow-up appointment scheduling but could occur in the same procedure with SIM lesion confirmation.

In this work, we conducted a non-significant-risk retrospective clinical study to assess the potential for SIM to facilitate biopsy characterization for a “see-and-treat” approach to localized ablation of prostate cancer (Figure 1) in 60 fresh core-needle biopsies from 39 patients undergoing MRI-US-guided diagnostic prostate biopsy. Leveraging a dual-stain H&E analog to emulate the appearance of standard histology slides [40] and a 20× objective lens to provide the necessary resolution for observation of clinically relevant prostate microarchitecture, we demonstrate that pathologist interpretation of biopsy images obtained on fresh diagnostic biopsies is feasible at a speed compatible with point of care decision making, and the method provides useful information to urologists in real time that could guide treatment decisions by effectively confirming or rejecting clinical impression of biopsy diagnosis based on medical imaging. However, imaging of fresh tissue in the absence of lengthy optical clearing steps, while maximizing the ability to image the entire surface of the biopsy, precludes imaging deeply into the sample to achieve sampling with depth. Therefore, it is possible that discrepancies in diagnosis or grading can occur between surface imaging with SIM and depth-resolved sampling with traditional permanent histopathology.

## 2. Materials and Methods

### 2.1. Instrumentation

The imaging of these samples was performed using a customized, multicolor SIM system utilizing a pattern projection unit based on a liquid crystal on silicon (LCoS) spatial light modulator (3DM, Fourth Dimension Displays, Dunfermline, Scotland) connected to an automated epifluorescence microscopy platform (RAMM, Applied Scientific Instrumentation, Eugene, OR, USA) using 30 mm cage system components (Thorlabs, Newton, NJ, USA). Illumination was provided by a multiline laser engine (LDI 6, Chroma, Bellows Falls, VT, USA), and excitation and emission wavelengths were discriminated using custom-manufactured multiband optical filters and beamsplitters (Chroma, Bellows Falls, VT, USA). We previously demonstrated this system’s ability to image biological tissues [23] and prostate biopsies [17] and resections [38,39]. This SIM system has a 1.3 mm × 1.3 mm single-frame field of view (FOV) with a 4.2-megapixel camera resolution. Images of entire biopsy surfaces were obtained by stitching together panoramas from multiple adjacent image frames of the fresh tissue. Optical sectioning using incoherent SIM was performed using the square-law demodulation algorithm described by Neil and colleagues [41]. Using a 20× objective lens (Nikon Plan Apo 20× 0.75 NA, Tokyo, Japan), the maximum attainable normalized spatial frequency is 0.21, corresponding to a measured optical section thickness of 6.2 mm. 

### 2.2. Sample Collection

All samples for this study were collected under the protocol approved by the Tulane University Biomedical Institutional Review Board and in accordance with all approved procedures (protocol code #2018-1243-TU and approval date of 11/4/2020). Eligible patients who were scheduled to undergo a transrectal or transperineal US-guided (+/− MRI fusion) diagnostic biopsy for suspicion of malignancy or active surveillance monitoring were consented for participation by research staff. Two additional 16-gauge core-needle biopsy samples were obtained from participants during the procedure: one from the ROI and another from the contralateral prostatic side and greater than 1cm from any targeted lesion, as determined using MRI-US fusion guidance (Philips UroNav, Amsterdam, Netherlands). Research biopsy cores were placed in prepared and labeled collection vials of 7.4 pH PBS immediately after excision by the urologist. Upon completion of the procedure, lab personnel retrieved the two samples and transported them to the nearby imaging lab for immediate processing. Participants in this study were aged from 49 to 78 years old with a mean of 65.3 years and a median age of 65 years. The patient population had PSA concentrations between 0.09 ng/mL and 24.4 ng/mL with a mean concentration of 7.9 and a median of 5.4 ng/mL. There were no patient-related adverse events associated with the additional core sample acquisition.

### 2.3. Staining

Samples were stained with the fluorescent dyes eosin Y and DRAQ5 to replicate the histological stain mechanism of hematoxylin and eosin. Prostate biopsies were individually submerged in 200 mL of a 50 mM solution of DRAQ5 (Biostatus, LTD, Leicestershire, United Kingdom) in PBS for three minutes, then immediately dipped in 1 mL of PBS to remove excess stain. Each biopsy was then submerged for 10 s in 50% w/v Alcoholic Eosin Y 515 (Leica, Wetzlar, Germany) in ethanol, then dipped repeatedly in 1% Surgipath Acid Alcohol (Leica, Wetzlar, Germany) to remove excess eosin Y stain. The fully stained biopsies were placed on a fresh KimWipe (Kimberly-Clark, Irving, TX, USA) for 3 s to absorb excess liquid from the biopsy. Finally, the biopsies were mounted individually between two 0.15 mm D × 24 mm W × 50 mm L coverglass slides with gentle pressure for imaging.

### 2.4. Imaging

Fluorescence images of the biopsy samples were obtained using the custom software-automated microscope. Eosin Y was excited at 528 nm and DRAQ5 was excited at 640 nm. Once the biopsy was properly mounted on the microscope system, the 528 nm laser was activated in the system software and a live feed of the sample was displayed on the computer’s monitor. Using the system’s motorized controls of the sample stage (X–Y-plane) and objective lens (Z-plane), the user utilized the live feed to determine the best initial focus position at the tissue surface, as well as the imaging dimensions required to image the sample surface completely. The same view was then used to optimize the power of each excitation laser to achieve the highest possible signal without saturating the camera detector. The imaging software automatically handled stage and objective lens movements to obtain multiple images of the tissue to reconstruct a mosaic. The sample stage moved in a serpentine pattern over the objective lens until the entire biopsy sample was imaged. To aid in imaging samples that did not have a microscopically flat surface, the user employed a custom autofocus routine. Autofocus was typically applied by re-focusing the objective lens every 4 frames with a step size of 4 μm in the Z-direction, using the 528 nm (eosin Y) channel only. The three patterned images were combined to generate a single SIM image for each frame [20]. The SIM images collected for both eosin Y and DRAQ5 were stitched together in Fiji [42] resulting in two grayscale images of in-focus information for each biopsy. A frame with strong features from each grayscale image was used to identify the appropriate pseudocoloring parameters. These parameters were then applied in a customized MATLAB script (version R2019b) to merge the two grayscale images and generate a new “pseudo-H&E” image as described previously [27,28].

Some samples were excluded from analysis based on technical failures. Specifically, samples with low DRAQ5 signal resulting in lack of nuclear visibility or high eosin Y signal resulting in camera saturation were excluded. Candidates for exclusion were identified via review by lab personnel and supported by statistical analysis.

### 2.5. Histopathology

Once imaging was complete, the biopsies were mounted in a histology cassette and submerged in 10% buffered formalin a minimum of 72 h before being sent for histology processing. A 4 µm section of each biopsy was cut, stained with hematoxylin and eosin, and mounted on a slide.

### 2.6. Imaging-Based Likelihood of Cancer Diagnosis

The urologist retrospectively analyzed the MRI-US fusion images and needle tracks for the research cores collected for this study. The urologist classified the likelihood of diagnosis of each core biopsy as (1) confident of non-malignancy, (2) equivocal for malignancy, or (3) confident of malignancy. ROI targeted PIRADS 4/5 lesions were classified as 3, PIRADS 3 lesions were classified as 2, and non-targeted systemic biopsies obtained from benign appearing regions of the MRI were classified as 1 on this ordinal scale. For purposes of analysis regarding a see-and-treat localized ablation setting, ratings of 2 (6 samples) were assigned a score of 1, which represents a conservative binarization approach. This provided, for each biopsy sample, a binary determination of clinician confidence in malignancy, in the absence of immediate histopathology confirmation. 

### 2.7. Masked Pathologist Review

Two board-certified anatomic pathologists were provided with the H&E slides and digital SIM images generated from the same prostate biopsy in sets of approximately 10 (ranging from 7 to 13) and masked to their correlation. Neither pathologist was provided any training images or materials for this study prior to review of the SIM images. The pathologists used an open-source web-based pathology image viewer based on HistomicsUI (Kitware, Inc., Clifton Park, NY, USA) to view and diagnose the SIM images. Binary diagnosis (malignant/non-malignant) and Gleason grade (if applicable), based on review of the investigational SIM images, were logged on a data collection sheet. Next, the pathologists looked at the H&E slides via their standard clinical microscope and documented diagnoses, Gleason grades (if applicable), and percent tumor on the slide (if applicable). Pathologist A deferred diagnosis on 8 of the samples (2 benign, 6 malignant as determined by H&E) and did not render a diagnosis—these were excluded from Pathologist A’s analysis. Pathologist B provided a final diagnosis for all images in the dataset. The pathology results from the diagnostic (non-research) cores were collected from the clinical pathology report as a reference for comparison against the targeted biopsies. 

### 2.8. Statistical Analysis

To confirm that the image quality of samples excluded from analysis due to technical failures was significantly different than those retained, a Kolmogorov–Smirnov two-sample test was used to compare the image intensity histograms between rejected and retained samples at an alpha value of 0.05. To quantify the pathologists’ results, a confusion matrix was constructed using their true positive, true negative, false positive, and false negative diagnoses. The deferred samples from Pathologist A are presented in the confusion matrix and identified as “indeterminate” but excluded from the subsequent accuracy calculations. The specificity, sensitivity, positive predictive value (PPV), and negative predictive value (NVP) were calculated, and the accuracy of SIM image diagnosis compared against each pathology rater’s own diagnosis on the H&E slides as the gold standard (the pathologist raters disagreed on the diagnosis of 1 sample). 

## 3. Results

**Figure 2:**
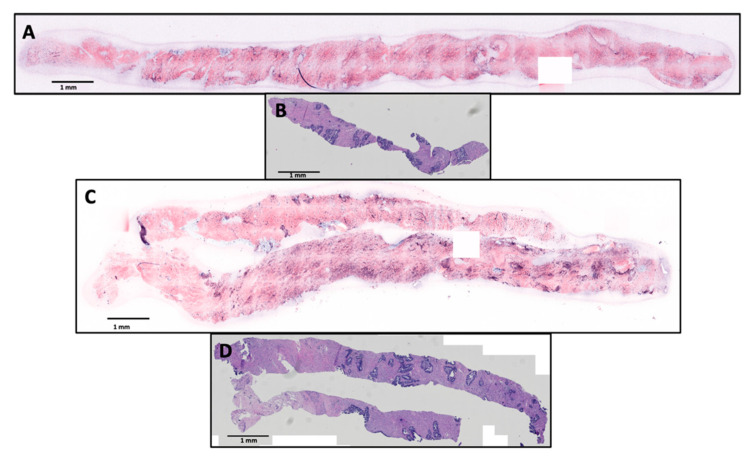
An example of a benign prostate biopsy imaged with SIM (**A**) and the subsequent histology slide (B) and an example of a malignant prostate biopsy with a Gleason score of 3 + 4 (**C**) and the subsequent histology slide (**D**). Images are shown to scale, all scale bars are 1 mm.

Seventy-eight (78) total biopsies were collected from patients but eighteen were not considered for inclusion in this study due to investigations into alternative imaging and staining parameters that made these samples inconsistent with the rest of the study (14), histology processing errors (3), and data save errors (1). Sixty (60) samples remained in this study, however fourteen were excluded for technical failures resulting in decreased image quality. The exclusions were validated by a two-sided Kolmogorov–Smirnov test on both fluorescent channels (*p* < 1 × 10^−6^). The pathologists analyzed the remaining 46 SIM images, and corresponding H&E slides obtained from the imaged biopsies. In this set, 31 samples were non-malignant and 15 were malignant. Figure 2 shows example SIM images and corresponding histology slides at scale of benign and malignant (Gleason 3 + 4, Grade Group 2) biopsies that were correctly diagnosed by both pathologist raters.

**Figure 3:**
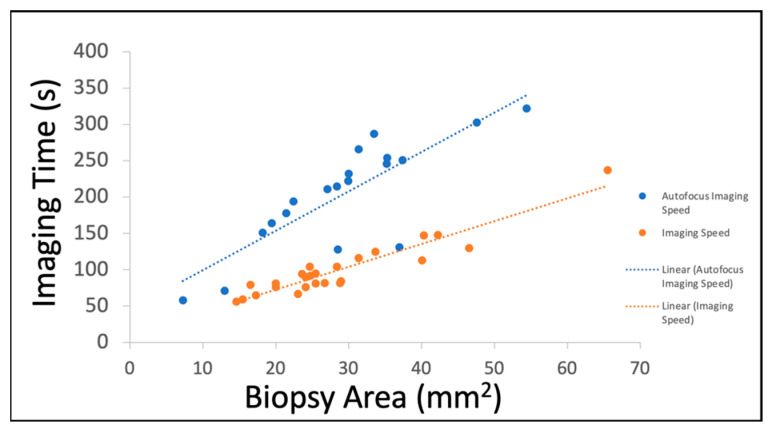
Imaging Time vs. Biopsy Area.

The processing time for each sample consisted of staining, imaging, processing, and reading. The time required for staining was 3 minutes with negligible time required to mount the sample on the system. The samples imaged with autofocus had an average area of 34 mm^2^ and an average imaging time of 199 s. The samples imaged without autofocus had an average area of 28.5 mm^2^ and an average imaging time of 99 s (Figure 3). Image processing takes approximately 4 minutes and is directly proportional to the frame count of the image. The pathologists reported reading each SIM image in 30 to 60 s. Cumulatively, these processing, imaging, and analysis steps can be completed well within 15 minutes, which is a commonly accepted timeframe for rapid on-site pathology assessments such as FSA and TPC.

In the 38-sample SIM image dataset diagnosed by pathologist A, the pathologist correctly classified 35 of 38 samples, for a total accuracy of 92%. In the 29 H&E-confirmed benign samples and 6 path-confirmed malignant samples, the same pathologist returned 6 true positive diagnoses and 29 true negative diagnoses, achieving a sensitivity of 67%, specificity of 100%, positive predictive value (PPV) of 100%, and a negative predictive value of 91% (Table 1). Pathologist B did not exclude any samples from the total dataset. Therefore, from the 46-sample SIM image dataset, pathologist B returned 9 true positive and 31 true negative diagnoses, resulting in a sensitivity of 64%, specificity of 97%, PPV of 90%, NPV of 86%, and overall accuracy of 87%. 

For pathologist A’s six true positive diagnoses, three of the Gleason scores were consistent with the corresponding slide and were all Gleason 3+4 (Grade Group 2). Two samples had lower Gleason scores on SIM than on the slides (Grade Group 1 vs. Grade Group 2, and Grade Group 2 vs. Grade Group 5), and one had a higher score from the SIM image than from the slide (Grade Group 2 vs. Grade Group 1). Pathologist B diagnosed nine true positives, eight of which had SIM–H&E Gleason grade alignment (four Grade Group 1 and four Grade Group 2) and one true positive had a lower Gleason score from the SIM image than the one from the histology slide (Grade Group 1 vs. Grade Group 2). Across both pathologists’ assessments, seven out of eight of the false negative diagnoses had primary Gleason patterns of 3, and the one that had a primary Gleason pattern of 5 was flagged as chronic inflammation by the pathologist rater on the SIM image.

**Figure 4:**
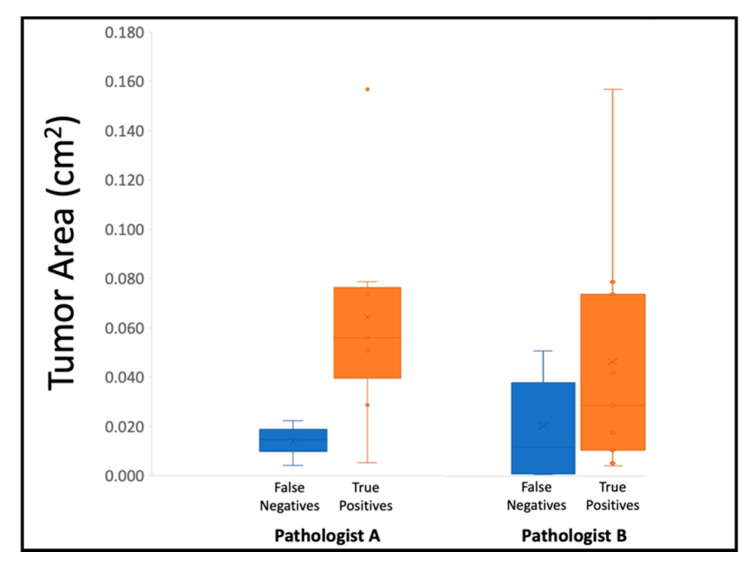
Tumor area on histology slides for false negative and true positive diagnoses on SIM. False negative calls based on SIM images were correlated with low tumor area in the final H&E sections, whereas true positive calls were correlated with higher tumor area.

It should be noted that SIM images of biopsies can vary from the histology slide due to intrinsic sample processing differences. Since the biopsies can be stained and imaged immediately after excision in their fresh state using SIM, without any embedding or sectioning, the resulting image typically represents the entirety of a single surface of the biopsy. However, the physical processing and sectioning required to create histology slides can be destructive to the 16-gauge biopsies, causing fragmentation of the tissue and reducing the area of the tissue delivered onto the slide. Thus, the relative assessment of fraction of one Gleason pattern over another in Gleason scoring can be affected by the total amount of tissue present in the image or slide. The sizes of the resulting pseudo-colored biopsy images ranged from 7.3 mm^2^ to 65.5 mm^2^ (71.3 megapixels to 842 megapixels). The non-destructive nature of SIM allows for the surface of a whole, intact biopsy to be imaged. Thus, the diagnostic area from one SIM image is typically much larger than a single section on an H&E slide. In this study, the area of the SIM images ranged from 2.2 times to 149 times larger than the subsequent histology section on the slides. This size difference contributes to tumor-presence variations between the SIM images and the histology slides. Figure 4 shows that lower tumor area on slides correlates to false negative SIM diagnoses and higher tumor area on slides correlates to true positive SIM diagnoses for both pathologists, indicating that the reason for false negatives on SIM was likely due to limited tumor content in the biopsy.

**Figure 5:**
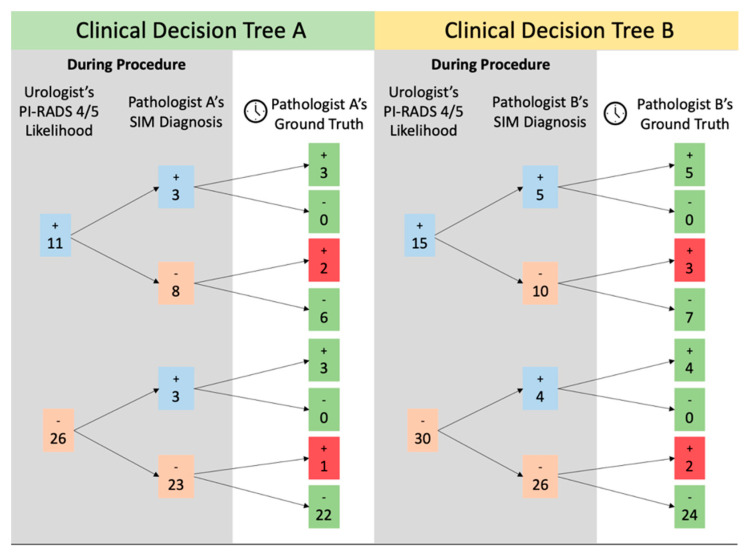
Decision tree of urologist’s interpretation of the likelihood of clinically significant cancer accuracy and subsequent SIM diagnosis accuracy. Both levels are compared to the pathologists’ “gold-standard” diagnoses from histology slides. The blue values represent the number malignant predictions or diagnoses, and pink values represent the number of nonmalignant predictions or diagnoses. Green numbers indicate correct diagnoses from the pathologist and red numbers indicate incorrect diagnoses from the pathologist.

When compared to diagnoses from the histology slides, the MRI-determined likelihood of clinically significant cancer resulted in 25 true negatives and 8 true positives and 5 false negatives and 7 false positives (1 sample could not be retrospectively analyzed because the patient data were no longer available to the urologist). These numbers were compiled into a “clinical decision tree” to represent information access and accuracy in a potential clinical setting (Figure 5). Of 30 samples with a sub-4/5 PIRADS rating by the urologist, 23 were also rated negative by pathologist A (96% correct) and 26 were rated negative by pathologist B (92% correct) based on SIM images obtained in real time. Conversely, three of these biopsies were correctly identified as malignant by pathologist A (100% correct) and four were correctly identified as malignant by pathologist B (100% correct). Similarly, if the urologist was suspicious of malignancy based on a 4/5 PIRADS score (15 samples), they were correct 53% of the time. Of this set, 6 out of 8 false positive predictions on MRI were determined to be benign by pathologist A, and 7 out of 10 false positives were confirmed benign by pathologist B. Pathologist A identified three out of three true positives in this set and pathologist B identified five out of five true positives. MRI images from one patient (true negative for pathologist A, false positive for pathologist B on SIM) were no longer accessible for retrospective analysis by the urologist, and thus this patient was excluded from the decision tree analysis.

## 4. Conclusions

In this work, a retrospective study to evaluate the potential for real-time bedside biopsy structured illumination microscopy to inform clinical decision making vis-á-vis immediate localized ablation was performed. A proposed decision tree based on pathologist review of SIM images demonstrates the potential impact of real-time biopsy imaging on a novel “see-and-treat” workflow for prostate cancer. In this assessment, on the individual biopsy level, pathologist A would have accurately confirmed suspicion of malignancy in 3 out of 11 samples and prevented the potential overtreatment from 6 samples when compared to just the urologist’s prediction. Additionally, they would have confirmed a non-malignant prediction in 22 of 26 biopsies and correctly flagged three patients for malignancy that would have been otherwise missed. Pathologist B would similarly have accurately confirmed suspicion of malignancy on-site in 5 of 15 samples and prevented potential overtreatment of 7 samples through an on-site checkpoint between urologist prediction and full histological workup. Of the 30 non-malignant predictions made on medical imaging alone in pathologist B’s dataset, 24 would have been accurately confirmed benign by SIM imaging, and 4 biopsies would be confirmed to have malignancy on-site. This approach would allow urologists to confirm their suspicions of malignancy and potentially commence treatment at the time of biopsy, instead of waiting for the processing required to make a diagnosis from a histology slide. Implementation of SIM on margins of ablation could have a clinically relevant application immediately. In a “see-and-treat” model of ablative treatment for clinically localized prostate cancer, high specificity (100% for pathologist A, 97% for pathologist B) would allow the treating physician to expand tissue margin to ensure more complete lesion coverage, thus improving oncologic outcomes. 

The accurate identification of Gleason grade from SIM images is important for discerning patient eligibility for localized treatment. The pathologists in this study were able to identify regions of malignancy with high consistency between Gleason scoring in both the SIM images and the histology slides. When the Gleason scores from the images and H&E slides did not align, the Gleason scores from the images underestimated Gleason scores from H&E slides for the most part, limiting risk of overtreatment due to SIM image interpretation. Since SIM imaging of the fresh tissues is non-destructive and H&E diagnoses can still be rendered as standard of care, permanent H&E can serve as a safety net for underestimations of malignancy at the time of biopsy acquisition. Urologists currently utilize imaging (US alone or MRI + US) and patient information to predict clinically significant prostate cancer (PIRADS 4 or 5), however, since this cannot be carried out with full confidence or confirmation, it cannot be used to educate on-site intervention. The addition of SIM diagnosis adds an extra, more accurate step in the decision tree of on-site intervention. In all instances, deferring to the diagnosis made via SIM adds valuable information and prevents potential overtreatment. Margins of the tumor are routinely underestimated on MRI, and thus ablative procedures generally overtreat the area due to unsure ablative zone edges. The possibility of real-time data with strong negative predictive capabilities allows for more accurate tumoral ablation while minimizing risk of erectile dysfunction or urinary incontinence during the procedure. MRI predictions based on PIRADS scoring on biopsy were accurate about half of the time (45%, 53%) compared to the 100% accuracy of subsequent positive diagnoses via SIM images (3, 5). Similarly, urologist negative predictions were accurate in 84% of cases (83%, 85%), whereas negative diagnoses obtained via SIM images were above 90% (96%, 92%).

The sizes of diagnostic prostate biopsies as well as the overall speed for the creation of digital pseudo-H&E images contribute to the compatibility of this process for point of care feedback. The area imaged by SIM for prostate biopsies ranges from 0.073 cm^2^ to 1.188 cm^2^ whereas the area of a single cut on the subsequent H&E slides ranges from 0.002 cm^2^ to 0.14 cm^2^. This means that SIM images give pathologists an average of 21 times more area in a single plane to evaluate. Histology slides do have the advantage of cutting the sample and being able to present multiple planes on a single slide. However, these are often incomplete areas of the sample due to the destructive processing and inherently delayed by required processing time. The imaging time is the highest source of variance for the total speed of the process. The association between the image size and time required for autofocus-utilized samples is less strong because the autofocus mechanism requires time to adjust the stage in the Z-direction and test the contrast. The imaging speed achieved with SIM is clinically relevant in both cases. The maximum time dedicated to imaging in this dataset is just over 6 minutes with the longest imaging times corresponding with autofocus-imaged samples. Image processing can be completed in 3 to 4 minutes depending on the number of frames comprising the imaging area. Currently, the step of creating a pseudo-colored image from the black-and-white images from the 528 nm and 640 nm SIM images requires the most time because the parameters are selected for each sample by study personnel. In the future, we hope machine learning will aid in this step and expedite the processing.

The future work of this study will involve the continued assessment of the clinical viability of SIM as a diagnostic tool toward developing a “see-and-treat" paradigm. The results presented in this paper are a promising preliminary study and affirm that this approach should be explored on a larger scale. To continue investigating SIM’s utility with diagnostic prostate biopsies, the technology needs to be applied to a larger set of specimens and presented to more pathologists for analysis. Combining SIM with rapid optical clearing steps, as demonstrated in [43], could perhaps improve sampling accuracy with regard to tissue depth in future studies. A limitation of this study was the deferral and exclusion rates created by inadequate autofocus, and limited 20× magnification SIM images of diagnostic prostate biopsies available for training the pathologist raters. As further studies are conducted and additional samples are obtained, investigation of SIM capabilities to improve upon the existing technique will be conducted to address the technical limitations identified in this initial study.

## Figures and Tables

**Table 1:** Confusion matrices for both pathologist raters and diagnostic results summary of SIM images against gold-standard histopathology slides. Positive predictive value is abbreviated PPV and negative predictive value is abbreviated NPV in this table. M stands for malignant and NM stands for nonmalignant.

Pathologist A					Pathologist B				
			H&E Slide					H&E Slide	
			Benign	Malignant				Benign	Malignant
			31	15				32	14
SIM	Benign	32	29	3	SIM	Benign	36	31	5
	Malignant	6	0	6		Malignant	10	1	9
	Indeterm.	8	2	6		Indeterm.	N/A	-	-
		**True Positives**	**True Negatives**	**Sensitivity**	**Specificity**	**PPV**	**NPV**	**Accuracy**	
Pathologist A		6	29	67%	100%	100%	91%	92%	
9 M, 29 NM									
Pathologist B		9	31	64%	97%	90%	86%	87%	
14 M, 32 NM									

## Data Availability

The data presented in this study is available within the article.
